# Bilateral Vestibular Hypofunction: Insights in Etiologies, Clinical Subtypes, and Diagnostics

**DOI:** 10.3389/fneur.2016.00026

**Published:** 2016-03-04

**Authors:** F. Lucieer, P. Vonk, N. Guinand, R. Stokroos, H. Kingma, Raymond van de Berg

**Affiliations:** ^1^Department of Otorhinolaryngology and Head and Neck Surgery, Division of Balance Disorders, Faculty of Health Medicine and Life Sciences, Maastricht University Medical Center, School for Mental Health and Neuroscience, Maastricht, Netherlands; ^2^Faculty of Health, Medicine and life Sciences, University of Maastricht, Maastricht, Netherlands; ^3^Service of Otorhinolaryngology and Head and Neck Surgery, Department of Clinical Neurosciences, Geneva University Hospitals, Geneva, Switzerland; ^4^Faculty of Physics, Tomsk State Research University, Tomsk, Russian Federation

**Keywords:** bilateral vestibulopathy, bilateral vestibular hypofunction, bilateral vestibular areflexia, bilateral vestibular loss, etiology, caloric tests, head impulse test, vestibular migraine

## Abstract

**Objective:**

To evaluate the different etiologies and clinical subtypes of bilateral vestibular hypofunction (BVH) and the value of diagnostic tools in the diagnostic process of BVH.

**Materials and methods:**

A retrospective case review was performed on 154 patients diagnosed with BVH in a tertiary referral center, between 2013 and 2015. Inclusion criteria comprised (1) imbalance and/or oscillopsia during locomotion and (2) summated slow phase velocity of nystagmus of less than 20°/s during bithermal caloric tests.

**Results:**

The definite etiology of BVH was determined in 47% of the cases and the probable etiology in 22%. In 31%, the etiology of BVH remained idiopathic. BVH resulted from more than 20 different etiologies. In the idiopathic group, the percentage of migraine was significantly higher compared to the non-idiopathic group (50 versus 11%, *p* < 0.001). Among all patients, 23.4% were known with autoimmune disorders in their medical history. All four clinical subtypes (recurrent vertigo with BVH, rapidly progressive BVH, slowly progressive BVH, and slowly progressive BVH with ataxia) were found in this population. Slowly progressive BVH with ataxia comprised only 4.5% of the cases. The head impulse test was abnormal in 94% of the cases. The torsion swing test was abnormal in 66%. Bilateral normal hearing to moderate hearing loss was found in 49%. Blood tests did not often contribute to the determination of the etiology of the disease. Abnormal cerebral imaging was found in 21 patients.

**Conclusion:**

BVH is a heterogeneous condition with various etiologies and clinical characteristics. Migraine seems to play a significant role in idiopathic BVH and autoimmunity could be a modulating factor in the development of BVH. The distribution of etiologies of BVH probably depends on the clinical setting. In the diagnostic process of BVH, the routine use of some blood tests can be reconsidered and a low-threshold use of audiometry and cerebral imaging is advised. The torsion swing test is not the “gold standard” for diagnosing BVH due to its lack of sensitivity. Future diagnostic criteria of BVH should consist of standardized vestibular tests combined with a history that is congruent with the vestibular findings.

## Introduction

Bilateral vestibular hypofunction (BVH) is a heterogeneous chronic condition characterized by a bilateral reduced or absent function of the vestibular organs, the vestibular nerves or a combination of both (1–3). Patients can report a variety of symptoms, such as oscillopsia, imbalance, visual vertigo, cognitive deficits, autonomic symptoms, and impaired spatial orientation. Depending on the etiology, neurological symptoms can also be present (e.g., ataxia), as well as auditory symptoms, such as hearing loss or tinnitus (4–7). BVH is an uncommon disorder and the estimated prevalence in 2008 was 28 per 100,000 US adults (8). The impact is still controversial and not always recognized. However, increasing evidence shows that BVH causes a high decrease in quality of life and imposes a high socio-economic burden due to work-related disabilities (9, 10).

Diagnosing BVH can be difficult and, therefore, BVH is often under- or misdiagnosed. Many challenges are met when establishing the diagnosis of BVH. Currently, many different diagnostic tests are used for vestibular evaluation, such as the caloric test, rotatory chair tests, (video) head impulse test (HIT), vestibular-evoked myogenic potentials (VEMP), dynamic visual acuity test (DVA), etc. (1, 4, 7). However, at this moment, no diagnostic standards regarding interpretation and implementation of vestibular test are available (7).

When the diagnosis of BVH is established in a patient, identifying the etiology still remains challenging. Previous studies showed that in 49–80% a definite or probable etiology could be defined, indicating that in 20–51% the etiology remained idiopathic (5, 11). The already identified etiologies of BVH vary tremendously (Table 1) (3, 12–27). The possible underlying causes of idiopathic BVH are still controversial. Studies suggest that migraine might play an important role and the prevalence of migraine has been shown to be higher in unclassified or idiopathic vertigo (28, 29). Furthermore, vestibular migraine is increasingly recognized as a cause of episodic vertigo (30). However, the relation between migraine and vertigo is not always recognized in clinical settings, since vertigo can precede headache, but may also begin with headache or appear later in the headache phase (28).

**Table 1:** **Etiologies of BVH (3, 31–33)**.

Idiopathic (51%)	
Toxic/metabolic (13–21%)	Antibiotics, furosemide, cisplatin, aspirin, alcohol, vitamine-B12 deficiency, folate deficiency, hypothyroidism, styrene poisoning, combination non-steroidal anti inflammatory drug + penicillin
Infectious (3.8–12%)	Meningitis/encephalitis/cerebellitis, Lues, Behçet, Borrelia, Herpes Simplex Virus, bilateral neuritis
Autoimmune (10%)	Cogan, Susac, Sarcoïdosis, Wegener’s, Sjögren, colitis, celiac disease, polyarteritis nodosa, antiphospholipid syndrome, other systemic diseases
Neurodegenerative	CANVAS, superficial siderosis, episodic ataxia, multiple system atrophy, polyneuropathy, SCA3, SCA6, hereditary sensoric and autonomic neuropathy type IV, other ataxias
Genetic	DFNA9, DFNA11, DFNA15, DFNB4, mutation chromosome 5q, 6q, 11q, 22q Muckle Wells (NLPR3)
Vascular	Supra- or infratentorial lesions, vertebrobasilar dolichoectasia
Neoplastic	Bilateral vestibular schwannoma, Neurofibromatosis type 2, metastasis lymphoma, malignant tumor
Trauma	Head trauma, iatrogenic (e.g., bilateral CI-implantation)
Other ear pathology	Bilateral Ménière’s disease, otosclerosis, bilateral labyrinthitis, cholesteatoma
Congenital/syndromal	e.g., CHARGE, Usher, Turner, enlarged vestibular aqueduct syndrome, Alport syndrome
Other	presbyvertigo, vestibular atelectasis, auditory neuropathy spectrum disorders, etc.

The variety in etiology of BVH is also reflected by different clinical manifestations of the disease. Currently, four different clinical subtypes have been described as follows: (1) recurrent vertigo and BVH: this clinical subtype is characterized by episodes of vertigo, followed by the development of symptoms of bilateral vestibular function loss. (2) Rapidly progressive BVH: patients experience a sudden onset or rapid progression in the course of BVH. (3) Slowly progressive BVH: the clinical symptoms of BVH develop gradually, mostly without episodes of vertigo. (4) BVH with neurological deficits: the clinical symptoms of BVH are combined with neurological deficits, such as peripheral polyneuropathy and/or cerebellar ataxia (4, 31).

Next to vestibular tests, many other tests, such as cerebral imaging, audiometry, and blood tests, can be used (on indication) in the diagnostic process. These tests do not evaluate the vestibular function and are mainly used to determine the etiology of BVH or coexisting problems (31). It is not yet established which tests should be part of the routine diagnostic work-up of BVH.

Overall, additional research is necessary to determine other possible etiologies of BVH and to fully understand the development and clinical course of BVH. The objective of this retrospective study was to further investigate the different etiologies of BVH, the different clinical courses of the disease, and the value of some diagnostic tests currently used in the diagnostic process of BVH. This information could be valuable in the establishment of diagnostic criteria for BVH.

## Materials and Methods

### Ethical Consideration

This study was performed in accordance with the guidelines outlined by Dutch legislation. According to the Medical Research Involving Human Subjects Act (WMO) ethical approval was not required due to the retrospective nature and anonymization of these data.

### Patients

A retrospective case review was performed on all patients diagnosed with BVH by, or under the supervision of, the senior author at the vestibular department of Maastricht University Medical Center between 2013 and 2015. It was confirmed that the patients met the following diagnostic inclusion criteria: (1) imbalance and/or oscillopsia during locomotion and (2) reduced responses (summated slow phase mean peak velocity of the nystagmus of less than 20°/s) during bithermal caloric tests. If a patient chart did not comprise all the essential information necessary to determine the etiology (including idiopathic etiology), the patient was excluded. Time of diagnosis was based on the date on which the vestibular tests were performed.

### Data Collection

Clinical records were reviewed as well as all qualitative and quantitative data concerning medical history taking, familial history taking (e.g., vestibular disorders, genetic disorders, etc.), vestibular tests (oculomotor tests, caloric tests, torsion swing tests, HITs), neurological examinations, and other diagnostic tests. Other diagnostic tests comprised pure tone and speech audiometry, laboratory tests (vitamin B12, folic acid, HbA1c, TSH, fT4, Borrelial serology, treponemal serology, and genetic tests), cerebral computed tomography, and magnetic resonance imaging. These data were collected in IBM SPSS Statistics 21. The non-vestibular diagnostic tests had only been performed on indication. Indications comprised an idiopathic etiology or an etiology requiring further diagnostic tests (e.g., an MRI of the cerebellopontine angle in Ménière’s disease to rule out other possible causes). The etiologies of BVH were classified into three groups: definite, probable, or idiopathic etiology (5).

The caloric tests were performed in all patients with water at 30 and 44°C. In 151 cases, the volume was 300 ml in 30 s. In three cases, the volume was uncertain, because the caloric test was performed elsewhere. Torsion swing tests were performed using sinusoidal rotation (0.11 Hz) with a peak velocity of 100°/s. An abnormal response was defined by a gain of <30%, based on the 95% confidence interval of the normative data obtained in the vestibular department of Maastricht University Medical Center. The horizontal manual HIT was considered abnormal when overt saccades were observed. The horizontal video head impulse was performed using the Eyeseecam system (EyeSeeCam VOG; EyeSeeCam, Munich, Germany). A video HIT was considered to be abnormal when pathological overt- and/or covert saccades were present in the raw eye traces and/or when patients showed a gain less than 0.6. Missing values from all diagnostic tests were reported as missing data and were not included in the analysis performed. Patients were diagnosed with Ménière’s disease using the diagnostic criteria for Ménière’s disease (34). The International Headache Society criteria for migraine were used to establish the diagnosis of migraine with or without aura (35).

### Statistical Analysis

Modules from IBM SPSS Statistics 21 were used for statistical analysis. The chi-squared test was applied to test for differences and correlations in nominal values. A significance level of *p* < 0.05 was chosen to determine significant differences within and across groups.

## Results

In this study, 154 BVH patients were selected. The mean age of the patients was 60 ± 12.5 years (range 19–85) at the time of diagnosis. In one patient, no exact date of assessment could be determined. Of the study population, 52.6% was male and 47.4% female. Imbalance was experienced by 98.1% of the patients and 68.5% complained of oscillopsia.

### Etiology

**Figure 1:**
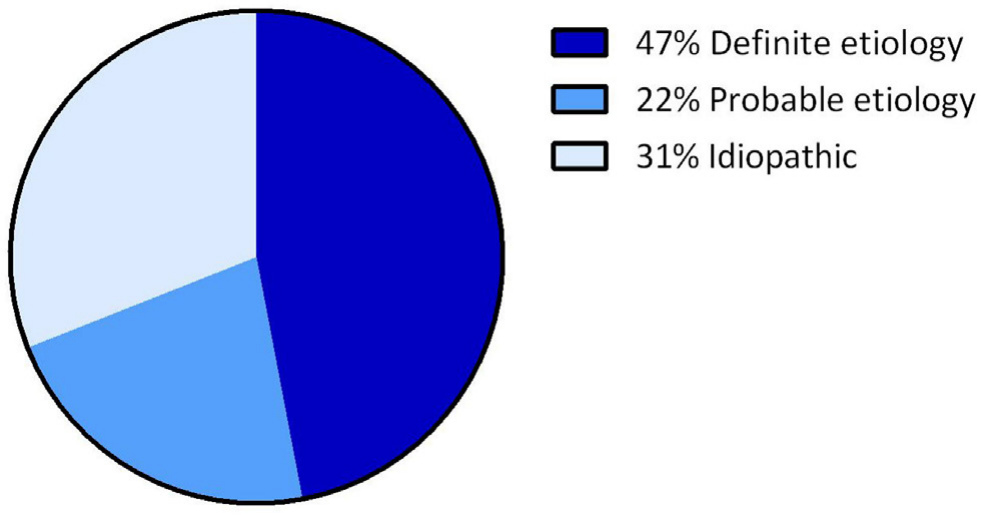
**Distribution of certainty of etiology**. A definite etiology was found in 47% of the BVH patients, a probable etiology in 22% of the BVH patients and in 31% the etiology of BVH remained idiopathic.

**Figure 2:**
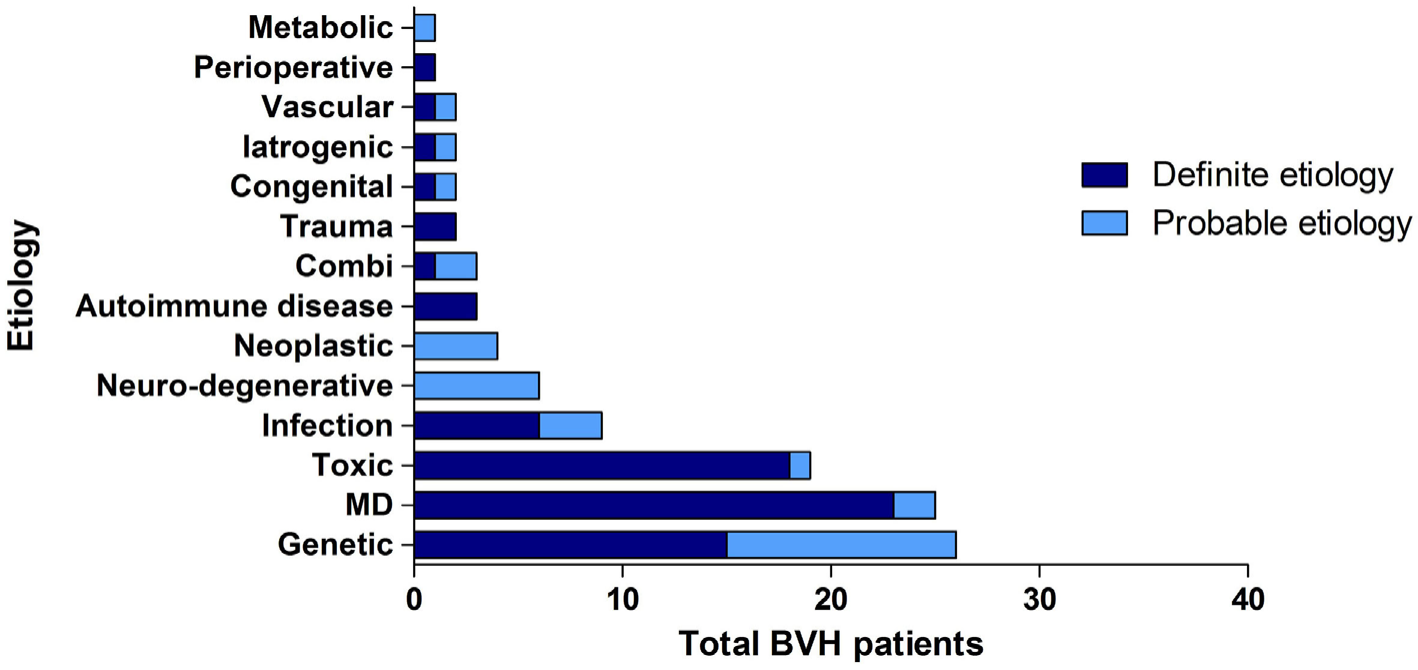
**Distribution of etiologies in the BVH group, divided into main groups of etiology and certainty of the etiology**.

Overall, the definite etiology of BVH was determined in 47% of the cases (*N* = 72) and the probable etiology in 22% (*N* = 34). In 31% (*N* = 48), the etiology of BVH remained idiopathic (Figure 1). In this study, BVH resulted from more than 20 different etiologies. The most frequent non-idiopathic etiologies were classified into genetic disorders (17%), Ménière’s disease (16%), ototoxicity (12%), infectious diseases (6%), and neurodegenerative diseases (4%) (Figure 2). The main groups and main observations will now be discussed.

Regarding genetic disorders, a definite diagnosis with a genetically confirmed mutation in the COCH gene was most prevalent (*N* = 15). In 11 patients, clinical presentation and family history were highly suggestive for genetic predisposition, but no genetic investigations were performed or no genetic mutation was found. In these patients, a genetic origin was, therefore, classified as the probable etiology.

Thirteen patients were diagnosed with bilateral Ménière’s disease and 12 patients with unilateral Ménière’s disease. In all 12 patients diagnosed with unilateral Ménière’s disease, a bilateral vestibular function loss was found. One patient experienced only one vertigo attack and one patient did not have objective hearing loss according to the diagnostic criteria of Ménière’s disease. Therefore, two patients were diagnosed as probable Ménière’s disease.

In the group of ototoxicity, BVH was related to treatment with Gentamycin in 14 patients. Two other cases resulted from treatment with Tobramycin and Neomycin and in two patients BVH developed after chemotherapy. In one patient, a probable ototoxic cause was found due to treatment with Quinine.

In the group of infectious diseases, BVH was definitely related to meningitis in childhood in six patients. Three other cases most likely resulted from Rubella, Epstein–Barr Virus, and Lyme’s disease. The group of neurodegenerative diseases consisted solely of six patients with the suspected diagnosis of cerebellar ataxia with neuropathy and bilateral vestibular areflexia syndrome (CANVAS). Strikingly, 23.4% of all patients (*N* = 36) reported an autoimmune disease in their medical history. Among them, three patients were identified with Cogan’s syndrome and only seven patients suffered from Ménière’s disease. In 3% (*N* = 5), a neoplastic abnormality was found. Four patients suffered from a unilateral vestibular schwannoma and one patient had a skull base meningioma. Head trauma caused BVH in two patients (bilateral skull base fracture and labyrinth contusion) and two patients were known with congenital abnormalities. In two patients, vascular abnormalities (cerebrovascular accident and after carotid endarterectomy) were found. In one patient, BVH developed after organ transplantation and in two patients after bilateral cochlear implantation. In one patient, renal failure was the probable cause of BVH. In three patients, a combination of two different causes resulted in BVH.

**Figure 3:**
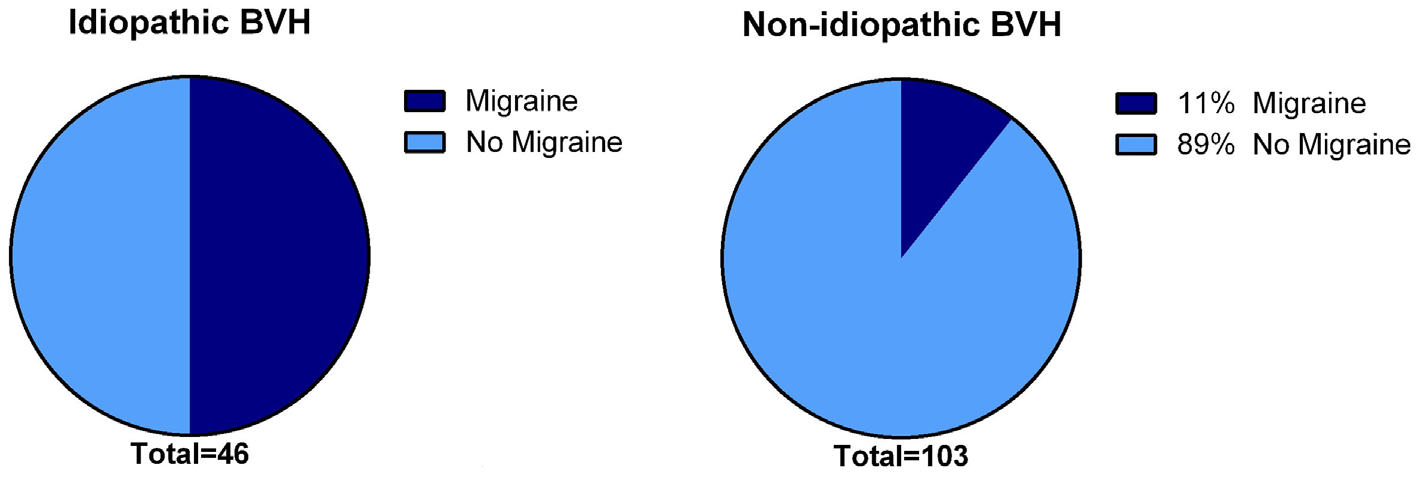
**Presence of migraine in the idiopathic BVH group versus the non-idiopathic BVH group**. In the idiopathic BVH group, migraine was significantly more present than that in the non-Idiopathic BVH group (50% versus 11%, *p* < 0.001).

In 31% (*N* = 48), the etiology of BVH remained idiopathic. The gender was equally distributed. Notably, the percentage of migraine among idiopathic patients was significantly higher compared to the group of patients with a definite or probable etiology of BVH (*p* < 0.001): 50% of the idiopathic BVH patients met the International Headache Society criteria (35) for migraine, in contrast to 11% in the non-idiopathic BVH group (Figure 3).

### Clinical Subtypes of BVH

**Figure 4:**
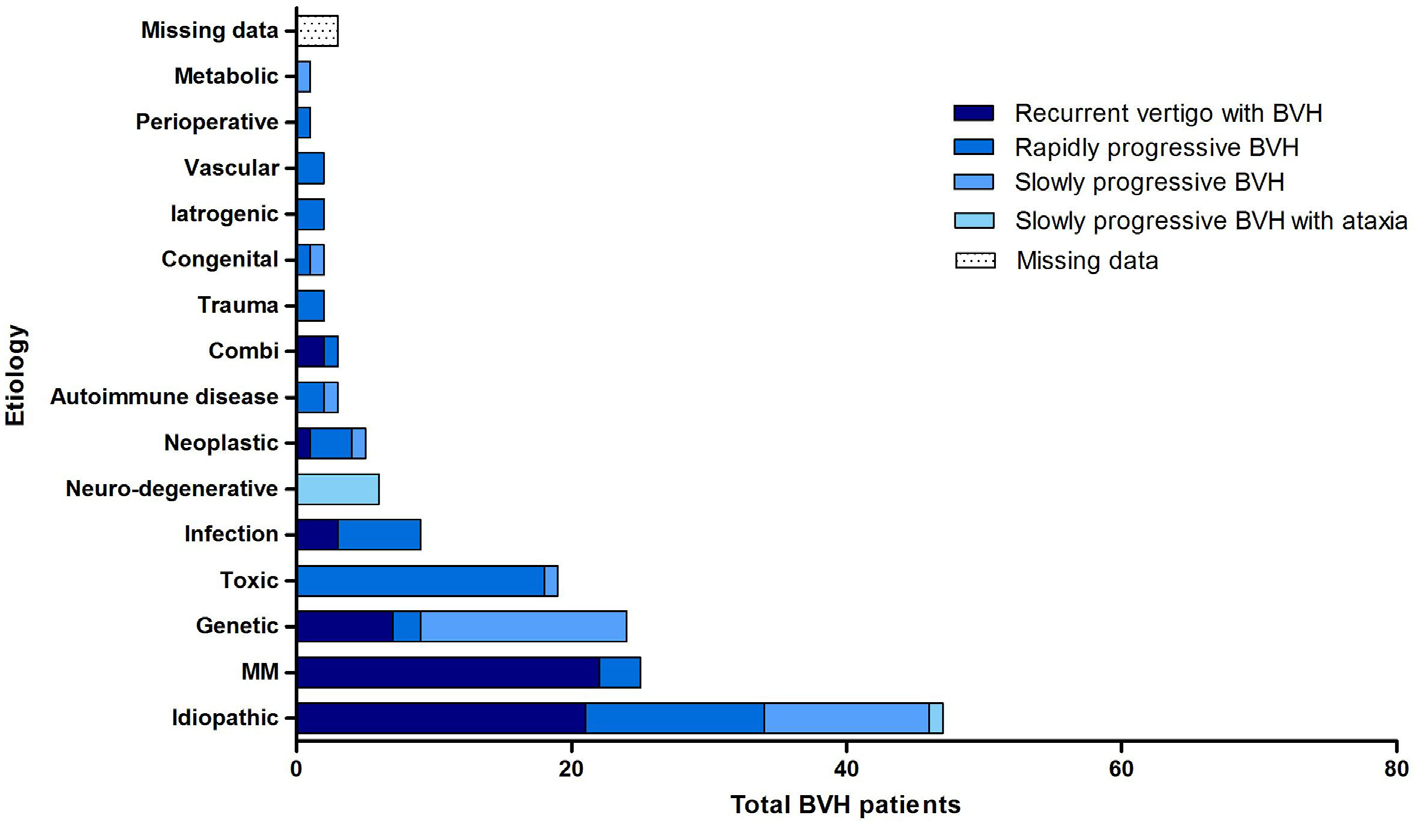
**Clinical subtypes of BVH with respect to the etiologies in the BVH group**.

All four previously described clinical subtypes were found in this population (Figure 4). Recurrent episodes of vertigo, followed by the development of BVH were found in 36.4% (*N* = 56) of all patients. Ménière’s disease (*N* = 23), genetic disorders (*N* = 7) and idiopathic BVH (*N* = 21) were most accounted for this clinical subtype. In 36.4% of the cases (*N* = 56), a rapidly progressive BVH was found. The main cause of this acute impairment was ototoxicity due to the adverse effect of medication, in particular aminoglycoside antibiotics (*N* = 14).

Thirteen patients with this clinical subtype belonged to the idiopathic group. BVH developed slowly progressively in 20.8% (*N* = 32) of the patients. In most cases, it comprised patients with a genetic disorder or patients from the idiopathic group. Slowly progressive BVH combined with neurological deficits was found in 4.5% (*N* = 7) of the cases. Six of these patients were suspected of CANVAS. Missing data arose in 1.9% of all cases.

### Diagnostic Tests

According to the inclusion criteria, bithermal caloric tests showed reduced responses with a summated slow phase velocity (SPV) of the nystagmus of less than 20°/s in all 154 patients. A mean of 6.3 ± 6.3°/s (range 0–19) was found. The HIT was abnormal in 109 out of 116 tested patients (94%). The seven head impulses that were considered to be normal, all involved manual HITs. In 38 patients, no data were found concerning the HIT. Data about the torsion swing test were present in 143 patients, of which 94 showed reduced responses (66%) (Table 2).

**Table 2:** **Results of torsion swing test and head impulse test**.

Torsion swing (N)	Normal	Abnormal	Not tested	Total
(V)HIT				
Normal	4	2	1	7
Abnormal	36	66	7	109
Missing data	9	26	3	38
Total	49	94	11	154

Pure tone and speech audiometry was performed in 143 patients. In 49% of these patients, there was normal hearing or only moderate hearing loss in both ears (modified Fletcher Index: 0–49 decibel). No hearing loss was found in 22 patients. In 36% of the patients, an asymmetric hearing loss was found. The results of the pure tone audiometry are presented in Table 3.

**Table 3:** **Results of pure tone audiometry**.

Impairment hearing loss	FI-high (dB)	Asymmetric hearing		Symmetric hearing	
		Best ear	Worst ear	Best ear	Worst ear
No impairment	0–19	17	0	28	21
Slight impairment	20–34	10	6	19	21
Moderate impairment	35–49	10	8	11	14
Significant impairment	50–64	10	3	9	3
Severe impairment	65–79	3	8	7	11
Profound impairment: including deafness	>80	5	30	14	18
Not tested	11				
Total patients	154	55	55	88	88

When indicated, patients were screened for abnormal levels of vitamin B12, folic acid, HbA1c, TSH, and fT4. Most strikingly, a vitamin B12 deficiency was found in 26 patients and a high folic acid was present in 27 patients. In eight patients, a combination of both findings was seen. Most cases involved a mild vitamin B12 deficiency, probably not related to the development of BVH. High levels of HbA1c were only found in patients already known with diabetes mellitus. In seven patients, abnormal thyroid function was found, of which three patients were known with thyroid dysfunction. In not one of these patients, a relation with regard to the development of BVH was suspected. An overview of blood test results is presented in Table 4. Approximately, one-third of all patients were tested for antinuclear antibodies (ANA) and anti-neutrophil cytoplasmic antibodies (ANCA). In six patients, a positive ANA, without a positive ANCA, was found. One of them was known with autoimmune disorders (rheumatoid arthritis, and diabetes mellitus type I) and one of them with Ménière’s disease. In the remaining four patients, the positive ANA was a new finding and they were referred to an immunologist. In only one of them, an underlying autoimmune disorder was suspected and the patient was treated with corticosteroids. In one patient, an atypical pattern of ANCA was found, without a positive ANA. This patient was already known with an autoimmune disorder (rheumatoid arthritis) (Table 5). Seventy-three patients were tested for antibodies against the Borrelia bacteria, of which five showed results of a previous Borrelial infection, without any signs of a recent infection or a history of typical complaints of neuroborreliosis. If necessary, a virologist was consulted. In two cases (3%), it was decided to still treat the patients against the Borrelial infection. Forty-one patients were screened for antibodies against the *Treponema pallidum* bacteria and no abnormalities were found (Table 5).

**Table 4:** **Blood test results in BVH patients**.

Results	Vitamin B12	Folic acid	HbA1c	TSH	fT4
Low	26^a^	1	–	2	–
Normal	39	32	44	60	56
High	–	27	4	3	2
Total	65	60	48	65	58
Not tested	89	94	106	89	96

*^a^Three patients were known with anemia in medical history due to Vitamin B12 deficiency*.

**Table 5:** **Results auto-antibodies and bacteria in BVH patients**.

Results	ANA	ANCA	*Treponema pallidum* (IgM)	*Borrelia bacteria* (IgM)	*Borrelia bacteria* (IgG)
Negative	50	51	41	71	66
Positive	6	0	0	2	7
Atypical	0	1	–	–	–
Total	56	53	41	73	73
Clinical implication	1	0	0	0	2
Not tested	98	102	113	81	81

Cerebral imaging studies were performed in 127 patients (*N* = 119 MRI; *N* = 8 CT) (Table 6). The following abnormalities were found: cerebellar atrophy (*N* = 4), cerebellar lesions (*N* = 3), unilateral vestibular schwannoma (*N* = 4, of which two patients did not have asymmetric hearing loss), supra- or infratentorial vascular lesions (*N* = 5), a skull base meningioma (*N* = 1), and abnormalities of the semicircular canals (e.g., fibrosis, calcification, inflammation, *N* = 4). No new abnormalities were found with computerized tomography. In three patients, abnormalities found with cerebral imaging were already expected due to previous findings on MRI or medical history. In 18 patients (14%), cranial imaging studies revealed new findings.

**Table 6:** **MRI and CT in BVH patients**.

		Type	*N* (%)
MRI	*Normal*		98 (63.6)
*N* = 119	*Abnormal*	Cerebellar atrophy	4 (2.6)
		Cerebellar lesions	3 (1.9)
		Unilateral VS	4 (2.6)
		Supra- or infratentorial vascular lesions	5 (3.2)
		Other	5 (3.2)
CT	*Normal*		8 (5.2)
*N* = 8	*Abnormal*		0 (0)
Not tested			27 (17.5)
Total			154 (100)

## Discussion

In this study, 154 patients were evaluated with regard to the different etiologies of BVH, clinical subtypes of BVH, and diagnostic tests to establish the diagnosis of BVH. Although the etiology of BVH could often be defined, 31% of the cases remained idiopathic. Remarkably, the prevalence of migraine in this idiopathic group was significantly higher compared to the non-idiopathic group. The different etiologies of BVH were partly reflected by the four clinical subtypes, as previously described (4, 31). Not all patients showed a pathological HIT or reduced responses with the torsion swing tests. Audiometry often contributed to the determination of the etiology (e.g., Ménière’s disease) and in this population, blood tests did not often contribute to the determination of the cause of BVH. Abnormal cerebral imaging was found in 21 patients.

### Etiologies

Patients were classified as having a definite, probable, or idiopathic etiology of BVH. Once etiology was not entirely certain or could not be confirmed with genetic research (e.g., CANVAS patients), etiology was described as a probable cause of BVH. Therefore, the amount of definite causes of BVH is probably a conservative estimation.

Genetic disorders and Ménière’s disease were the most frequent non-idiopathic causes of BVH in this population. Regarding genetic disorders, most patients presented with a mutation in their COCH gene. This mutation has been identified to cause autosomal dominant non-syndromic hearing loss accompanied by vestibular disorders (DNFA9) (13). Due to the high prevalence of DNFA9 in the Netherlands and Flanders (36), it is possible that the presence of DFNA9 was higher in this population of BVH patients. Although a strong underlying familial character seems to be present in multiple vestibular disorders, genome-wide association studies remain very difficult to perform, partly due to the clinical heterogeneity of vestibular disorders (12, 37). Therefore, BVH patients suspected of a genetic disorder (based on clinical manifestation and family history) other than a confirmed COCH gene mutation, were classified as having a genetic disorder as the probable cause of BVH.

Regarding Ménière’s disease, a unilateral Ménière’s disease was found in 12 cases, in which no definite etiology could be determined to explain the vestibular hypofunction of the “unaffected” side. In some cases, bilateral Ménière’s disease was suspected on history, but no objective hearing loss could be defined with pure tone audiometry. However, other cases did not report any cochlear symptoms on the “unaffected” side. It probably shows that the vestibular system can (already) be affected bilaterally, while cochlear symptoms are present on only one side. This is in line with a previous study that showed that abnormal VEMP’s were present in 27% of the “unaffected” ears in unilateral Ménière’s disease (38). This bilateral vestibular involvement in a clinically unilateral Ménière’s disease has also important clinical implications: it shows that the use of diagnostic parameters, such as the interaural asymmetry in calorics and VEMP’s is not sufficient for diagnostic purposes in Ménière’s disease (3, 31, 38, 39).

Furthermore, when intratympanic gentamicin treatment is considered for intractable unilateral Ménière’s disease (40), an ENG should always be performed to determine the vestibular function of the “unaffected” side. If not performed, this could imply a risk of inducing an even more severe BVH.

Neurodegenerative diseases and ataxia were found in only seven patients, which varies tremendously compared to earlier studies (5, 11). This finding could imply that the distribution of the etiologies of BVH depends on the clinical setting in which patients are seen. In this study, patients were selected at the vestibular department of the Department of Otorhinolaryngology and Head and Neck Surgery in Maastricht University Medical Centre, whereas in previous studies patients were selected in neurological departments (5, 11). This could explain the low percentage of BVH with neurological deficits and might also be the reason why fewer patients showed a slow progression of BVH, which is usually seen in patients with neurological disorders (20). Overall, the “real” BVH population is probably a reflection of both study populations.

A unilateral vestibular schwannoma with bilateral vestibular loss was found in four patients. This is an atypical finding, since BVH is mainly found when schwannomas appear bilaterally as in neurofibromatosis type II (41), not unilaterally. To our knowledge, literature has not reported about this type of finding. However, most studies about schwannomas report on labyrinthine asymmetry in calorics, without incorporating the absolute values (42–46). This increases the chance of missing a symmetric low vestibular response (3). A possible explanation for the vestibular loss on the contralateral side could be an endolymphatic hydrops on this side (47). Nevertheless, regarding the etiology of vestibular hypofunction on the side without a schwannoma, a definite cause could not be established, although in one out of the four cases, vestibular symptoms worsened after treatment with Gamma Knife.

Among all 154 BVH patients, 23.4% were known with autoimmune disorders in their medical history. Only in the three patients with Cogan’s syndrome, a definite causal relationship could be identified between BVH and autoimmunity. Seven of the patients with a known autoimmune disorder suffered from Ménière’s disease, resulting in a possible link between BVH and autoimmunity (48, 49). However, the percentage of autoimmune disorders in western countries is only around 8% of the total population (50), in contrary to the 23.4% found in this population. Therefore, autoimmune disorders might not always cause BVH directly, but could probably play a modulating role in the development of the disease.

In 31%, the etiology of BVH remained idiopathic. This differs from previous results in a large relatively recent study, in which in approximately 51% no definite or probable etiology of BVH could be defined (5). This difference could partially be explained by the increased knowledge regarding the underlying cause of BVH, the relatively high prevalence of genetic disorders in this population and the different clinical settings in which patients were seen. Notably, 50% of the idiopathic patients reported a history of migraine headaches according to the criteria of the International Headache Society. This was significantly higher than that in the non-idiopathic group and higher than would be expected in the normal population: migraine normally affects only 8% of males and 17% of females (51, 52). Next to the link between migraine and vertigo in vestibular migraine (30, 35), a suggestive linkage between migraine and BVH has been proposed before (4, 53). This study seems to confirm that migraine might play a significant role in the idiopathic variant of BVH and it shows the importance of screening for migraine features in the diagnostic process of BVH. In the future, additional studies could focus on the role of migraine in BVH and, in particular, the non-classical manifestations of this disease.

### Clinical Subtypes

Most non-idiopathic BVH patients presented with a clinical subtype that would be expected with respect to their etiology (e.g., mainly rapidly progressive symptoms after ototoxic medication). Among the idiopathic BVH patients, all four different clinical subtypes were reported, showing an overlap between the clinical presentations of non-idiopathic and idiopathic BVH. This is one of the contributing factors of the challenging diagnostic process of BVH (3, 4, 31).

### Vestibular Tests and Inclusion Criteria

#### Caloric Test

Inclusion criteria comprised reduced responses during bithermal caloric irrigations. However, absent responses during bithermal caloric irrigations do not necessarily indicate a complete loss of the vestibular function (7). Therefore, symptoms had to match caloric findings in order to get a more specific selection of patients. In addition to that, other vestibular tests were evaluated.

#### (Video) Head Impulse Test

In contrast to previously proposed criteria (7), a positive HIT was not included in the inclusion criteria due to the following reasons: (1) a bilateral pathological HIT was previously found in only 73% of a BVH population, suggesting that a normal HIT does not necessarily rule out a vestibular deficiency (1). (2) A manual HIT observed with the naked eye of experts is false negative in about 50% of the patients, when compared to the VHIT (31). In this study, all HITs performed with a VHIT-system showed pathological responses, in contrary to some manually performed HITs. Since a manual HIT can be false negative, it cannot be ruled out that (some of) the manual HITs in this study were actually false negative ones. Nevertheless, around 94% of all HITs corresponded with the findings during bithermal caloric irrigations. Taking these findings into account for the future diagnostic process of BVH, we would strongly suggest performing a VHIT instead of a manual HIT.

#### Torsion Swing Test

Although the torsion swing test is sometimes considered to be the “gold standard” for BVH (3), it was not included in the inclusion criteria since it is very specific, but seems to have a lower sensitivity, leading to false negative results (7, 31). In this study, only 66% of the tested patients showed an abnormal response with the torsion swing test at 0.11 Hz, implying a possible lack of sensitivity. The preservation of vestibular responses in the torsion swing test can possibly be explained by two hypotheses. (1) The torsion swing test stimulates the vestibular system closer to the optimum frequency sensitivity of the semicircular canals than the caloric test and the HIT (54, 55). When vestibular function would decline evenly distributed between frequencies in BVH, the vestibular system is most likely to longer preserve its response around the frequencies with optimum sensitivity. (2) In contrast to the caloric test and HIT, torsion swing testing is most likely to have more input from the otolith system (7). Since VEMP’s and DVAs were performed in only the minority of patients, they were not used in the total analysis. Therefore, inputs from otoliths during the torsion swing test could not be ruled out in this BVH population. For the future diagnostic process of BVH, torsion swing testing can be used complementarily (e.g., measure severity of BVH when the caloric test and HIT show no responses anymore) but not as the only test to diagnose BVH (31, 56).

#### Limitations of This Study Regarding Vestibular Tests

Inclusion criteria comprised vestibular symptoms combined with reduced responses during bithermal caloric tests. The selected patient group does, therefore, probably not reflect the whole BVH population, since the caloric test can miss, e.g., selective mid- to high-frequency loss of canals or isolated dysfunction of the otoliths. Depending on the diagnostic criteria for BVH, this leads to a lower sensitivity (7, 57). Next to this, specificity could be compromised by the fact that reduced caloric responses not always indicate a true vestibular loss. Uncontrollable factors, such as anatomy of the temporal bone could lead to reduced temperature conduction and as a consequence lead to false-positive results (58). However, since no diagnostic criteria for BVH are available yet and VEMP’s and DVAs were only performed in the minority of patients, it was decided to only use the caloric test as a parameter for the vestibular function. Nevertheless, VEMP’s, DVAs, and motion perception might play a more important role in the future diagnostic process of BVH (7, 31, 59–62) and our research group is, therefore, prospectively testing these parameters in BVH patients (not yet published).

### Other Diagnostic Tests

Currently, many other diagnostic tests are used for determination of coexisting problems or the etiology of BVH (31). Some of them will be briefly discussed below.

#### Audiometry

Audiometry often contributed to the determination of the underlying etiology (e.g., Ménière’s disease) of BVH and seems to be a valuable diagnostic tool when indicated. Considering otoacoustic emissions were not performed in these BVH patients, auditory neuropathy could have been missed during the determination of the etiology. Nevertheless, speech audiometry was performed on these patients, and as poor speech recognition is also a criterion of auditory neuropathy disorder, this obstacle could partially be overcome by these tests (32, 63–65). Notably, in approximately half of the patients, no to moderate hearing loss was found by using pure tone and speech audiometry. It shows the importance of hearing preservation during vestibular implantation, in case a vestibular prosthesis would become a clinically useful therapeutic device in the future (66–71).

#### Blood Tests

In this study, blood tests results of vitamin B12, folic acid, HbA1c, TSH, and fT4 showed that in case of abnormal blood results, no strong clinical implications were found with regard to the diagnosis or treatment of BVH. Limitation of this retrospective study was the fact that patients were referred to their general practitioner or other specialists for further diagnostics and follow-up of their vitamin B12 deficiency and high levels of folic acid. Not all data could be obtained. Nevertheless, the use of some of the blood tests (HbA1c, TSH, fT4) could be reconsidered in the diagnostic trial of BVH.

Although autoimmunity might play a role in the pathogenesis of some BVH subpopulations (72), the use of routine ANA and ANCA antibodies yielded little in this population. Seven patients showed positive results for ANA or ANCA, but some of the ANA positive results might be attributed to the fact that ANA positivity is also found in a certain percentage of normal individuals (73). The positive ANA in the patient with Ménière’s disease could be explained by a relation between Ménière’s disease and autoimmune diseases, described in earlier studies (48, 49, 74). Eventually, only 1 out of 157 tests had a clinical consequence: the patient was treated for a suspected autoimmune disorder. Therefore, in the future diagnostic process of BVH, routine testing of ANA and ANCA could probably be redundant and might be reserved for more specific cases with a higher suspicion of an underlying autoimmune disorder. However, the routine use of inner ear-specific auto-antibodies should still be investigated (72).

Two out of 73 patients tested for Borrelia were eventually treated for a Borrelial infection. The relation between BVH and Borrelia remained questionable in these cases due to the absence of typical complaints of neuroborreliosis and the fact that it can be a diagnostic challenge to prove that a neurological manifestation has been caused by Borrelia (75). This is due to the low positive predictive value of the test. Still, the negative predictive value is very high (76, 77). Although only a few cases were suspected of BVH due to a Borrelial infection, it should be noted that Borrelia is a non-endemic infectious disease in the demographic area in which patients were selected for this study (78). Therefore, blood tests to identify infection with the Borrelia bacteria might still be important, especially in areas were the bacteria is active.

Neurosyphilis has also a low incidence in the Netherlands, partially explaining the absence of antibodies against the *Treponema pallidum* bacteria in this BVH population. Nevertheless, screening for Treponema can still be considered in risk groups and in areas where treponemes are known to be endemic (79, 80).

#### Cerebral Imaging

Cerebral imaging studies revealed new findings in 18 patients, which is less compared to previous studies (5, 81), but this is again probably related to the study group. Cerebral imaging has shown to play an important role in the diagnosis of BVH, reflected by the new findings detected by MRI. In addition to that, cerebral imaging is also inherent to some diagnostic work-ups (e.g., an MRI in Ménière’s disease to rule out other possible causes) (34). Therefore, in the future diagnostic process of BVH, cerebral imaging should be performed in cases with a less clear etiology of BVH as well as within the framework of diagnostic work-ups. In most cases, MRI would be the preferred modality.

## Conclusion

Bilateral vestibular hypofunction is a heterogeneous condition with various etiologies and clinical characteristics. Migraine seems to play a significant role in idiopathic BVH and autoimmunity could be a modulating factor in the development of BVH. The distribution of etiologies of BVH probably depends on the clinical setting. In the diagnostic process of BVH, the routine use of some blood tests can be reconsidered and a low-threshold use of audiometry and cerebral imaging is advised. The torsion swing test is not the “gold standard” for diagnosing BVH due to its lack of sensitivity. Future diagnostic criteria of BVH should consist of standardized vestibular tests combined with a history that is congruent with the vestibular findings.

## Author Contributions

All authors contributed extensively to the work presented in this paper. FL and PV wrote the manuscript. RB supervised the writing and edited the manuscript. HK supervised the writing, reviewed the manuscript, and edited the manuscript. NG and RS reviewed the manuscript and edited the manuscript.

## Conflict of Interest Statement

The first author was supported through funding of MedEl. The remaining coauthors declare that the research was conducted in the absence of any commercial or financial relationships that could be construed as a potential conflict of interest.
